# Supplementary material for: Translators as mediators to mend the psychological gap between source text and target text: A corpus-based study on the Chinese English translation of modal verbs in the Chinese Report on the Work of the Government (2000–2022)
Source: PLoS One. 2025 Mar 28;20(3):e0320490. doi: 10.1371/journal.pone.0320490 (PMC11952205; doi:10.1371/journal.pone.0320490)
Supplement: Attachment — Submitted filename: Response to Reviewers.docx [file pone.0320490.s002.docx]

**Response to Reviewer 1**

**Reviewer #1:** The manuscript (PONE-D-24-48903) deals with the rendering of modal verbs in Chinese Government Work Reports from 2000 to 2022, exploring how to bridge the psychological gap between ST and TT. There are a relatively detailed, comprehensive literature review, a clear research methodology, a sound analysis, and a reliable conclusion. The article will be improved in quality if the author(s) can summarize the achievements on the C-E translation of the Reports and modal articles, and improve the language and style. On the one hand, these achievements can indicate whether the present study is original and can help strengthen the depth of analysis; on the other, the linguistic and stylistic improvements ensure the normativity of the article as a piece of scholarly work.

**Response:** Thank you for your thorough review and constructive suggestions for my manuscript PONE-D-24-48903. I appreciate your recognition of the paper's strengths and your valuable recommendations for improvement.

In response to your suggestions, I have made substantial revisions to enhance the manuscript's quality. Regarding the achievements in C-E translation of Reports and modal articles, I have significantly expanded the literature review section to include both seminal works and recent studies. Notable additions include Zhang and Liu's (2020) diachronic study of translation strategies, Chen and Wang's (2022) research on translation memory systems, and Liu et al.'s (2023) analysis of modal verb translation patterns. These additions not only provide a more comprehensive overview of existing achievements but also clearly demonstrate our study's originality in examining translators' mediating role through modal verb translations.

Concerning linguistic and stylistic improvements, I have thoroughly revised the manuscript to enhance its scholarly presentation. The revisions include more precise academic language, improved structural coherence, and refined argumentation throughout all sections. These modifications have strengthened the paper's academic rigor while maintaining clarity and accessibility.

I believe these revisions have substantially improved the manuscript's quality and hope they address your concerns satisfactorily. Thank you again for your valuable feedback.

**Response to Reviewer 2**

**Reviewer #2:** This study investigates the mediating role of translators in bridging the psychological gap between the source text (ST) and the target text (TT) through a corpus-based analysis. It specifically examines the translation of modal verbs in Chinese Government Work Reports (GWRs) from 2000 to 2022. The study is interesting and well-written, employing an appropriate methodology. However, the following areas require further attention:
1. Insufficient Review of Previous Studies: The theoretical framework needs to be strengthened by incorporating more relevant studies from existing literature.

**Response:** Thank you for your constructive feedback on our manuscript. I have carefully addressed your concerns and made substantial revisions to strengthen the paper. Regarding the theoretical framework, I have significantly expanded the literature review by incorporating additional relevant studies of over 400 words. Specifically, I have added comprehensive reviews of research applying Halliday's (2004, 2010) modal value system and studies utilizing Jiang and Yang's (2013) and Xu's (2018) classifications of Chinese modal verbs. This enhancement provides a more robust theoretical foundation for our research and better contextualizes our study within existing scholarship.

2. Underdeveloped Discussion Section: The discussion should be expanded to include a comparison of the results with findings from both theoretical and empirical studies.

**Response:** As for the discussion section, I have thoroughly revised and expanded it from approximately 500 to 750 words. The enhanced discussion now includes detailed comparisons between our findings and both theoretical and empirical studies in the field. I have incorporated insights from recent research by Li and Pan (2021) on translators' role as cross-cultural mediators, House's (2015) work on cultural filtering in translation, and Schäffner's (2004) perspectives on political discourse translation. These additions provide a more comprehensive analysis of our results within the broader context of translation studies. I believe these revisions have significantly strengthened the manuscript and hope they adequately address your concerns.

**Response to Reviewer 3**

**Reviewer #3**: This manuscript investigates the role of translators as mediators, focusing on the psychological gap between the source text (ST) and target text (TT) in the context of modal verbs in Chinese-to-English translations of government work reports (2000–2022). Employing a corpus-based approach, the study offers insightful observations on linguistic patterns, translation strategies, and the socio-cultural implications of modal verb usage.

The research aligns with current interests in corpus linguistics, translation studies, and discourse analysis, making a valuable contribution to the field. The corpus-based methodology is well-suited for exploring linguistic and translation patterns systematically. I strongly recommend this article for publication with a minor changes.

1. Merge Introduction and Literature Review:
The introduction and literature review have overlapping content. Merging them into a single section will streamline the structure and reduce redundancy.

**Response:** Thank you very much for your thoughtful and encouraging feedback on my manuscript. I greatly appreciate your recognition of its contribution to the fields of corpus linguistics, translation studies, and discourse analysis. Your acknowledgment of the value of our corpus-based methodology and its relevance to exploring linguistic and translation patterns is truly gratifying. As of your suggestion to merge the Introduction and Literature Review, I have carefully considered your recommendation and have made the following revisions accordingly: 1. I have reorganized the structure of these two sections: I have moved the research questions from the Introduction to the Literature Review section. This change allows the research questions to emerge more naturally from literature, providing a stronger theoretical foundation for the study. 2. I have refined the Introduction section: The Introduction has been streamlined to present the key focus of the study more directly and concisely. Redundant overlaps with the Literature Review have been eliminated to ensure clarity and cohesion. 3. I have expanded the Literature Review section: I have enriched the Literature Review with additional relevant studies, making it more comprehensive and systematic. It now provides a thorough exploration of modal verb translation, the mediator role of translators, and existing scholarship on translating Chinese government work reports. 4. I have also improved academic writing style: I have revised the language throughout the manuscript, ensuring it adheres to academic norms with improved precision, clarity, and professionalism.

2. The manuscript would benefit from referencing more recent and relevant studies to strengthen its theoretical framework. I recommend citing the following works:

**Response:** Regarding your suggestion to strengthen the theoretical framework, I have significantly expanded the literature review by incorporating additional relevant studies, including Halliday’s (2010) modal value system and the classifications of Chinese modal verbs proposed by Jiang and Yang (2013) and Xu (2018). These additions provide a more robust theoretical foundation and better contextualize our study within existing scholarship. Moreover, I have carefully read the articles you suggested and included the following three in the revised manuscript: Hu and Afzaal (2024), which highlights the use of multilingual corpora in political text translation, offering insights into the value of corpus-based approaches; Liu and Afzaal (2021), which explores syntactic complexity in translated and non-translated texts and supports our discussion on simplification strategies in political translation; and Afzaal and Du (2023), which addresses syntactic complexity in translated eHealth discourse and informs our analysis of cross-linguistic variations in specialized texts such as political discourse. These references have been integrated into the manuscript to strengthen its theoretical and methodological foundation. I sincerely appreciate your insightful suggestions, which have greatly enhanced the quality of the manuscript.

**Response to Reviewer 4**

**Reviewer #4:** Dear Authors,
I have carefully read the manuscript and find that it shows some originality in examining the psychological gap between source and target texts through modal verb analysis, with a well-designed corpus method and a rigours theoretical framework. The manuscript is well-written and is of good readability. However,
1. The findings section (Part 4) requires some revisions and elaborations. Some citations of existing literature appear superficial and lack sufficient engagement with the cited works. For instance, when referencing Nida (2001), Baker (2011), and Halliday (2010), the paper would benefit from more elaborations on how these scholars’ perspectives specifically relate to your findings.

**Response:** Thank you very much for your thoughtful and constructive feedback on the manuscript. Regarding the findings section (Part 4), I have carefully revised and elaborated on this part as per your suggestion. The references to Nida (2001), Baker (2011), and Halliday (2010) have been expanded to establish stronger links between these scholars’ perspectives and our findings. This enhancement ensures that the citations are more substantive and directly relevant to the discussion, thereby better contextualizing our study within the broader academic framework.

2. The writing style of this paragraph is of monotonous structure, and the numbered list format creates a mechanical presentation that lacks cohesion between ideas. The content should be reorganized into a cohesive paragraph with natural transitions between ideas. For example,
Third, the observed modal value shifts have several implications for the translation process and its impact on the TT: 1. By translating high-value modal verbs into medium and low-value equivalents, translators create a more flexible and reader-friendly tone in the English versions of the GWRs. This adjustment may make the reports more palatable to an international audience, potentially reducing the psychological distance between the government and its readers (Nida, 2001). 2. The shift from predominantly high-value modal verbs in Chinese to a more balanced distribution in English reflects an awareness of cultural differences in expressing authority and commitment. This adaptation demonstrates the translator's role as a cultural mediator (Hatim & Mason, 1990). 3. While the literal meanings of modal verbs may change, these shifts often aim to maintain pragmatic equivalence, ensuring that the intended effect of the original text is preserved in the translation (Baker, 2011). 4. The more varied use of modal values in the English translations may encourage greater reader engagement by presenting information in a less absolute manner, allowing for more nuanced interpretation (Halliday, 2010).

**Response:** Thank you for pointing out the mechanical presentation and lack of cohesion in certain sections. In line with your feedback, I have reorganized the content, including the part you highlighted, into cohesive paragraphs with natural transitions. The revised structure avoids numbered lists and employs a more sophisticated and engaging narrative style. This adjustment not only improves the readability of the manuscript but also enhances its academic tone and fluidity.

3. There are some minor tweaks in grammar:
(1) Use the full forms of abbreviations in the title: ST (source text), TT (target text), C-E (Chinese-to-English)
(2) This study aims to explore how translators function as mediators in this process by focusing specifically on the translation of modal verbs in Chinese government work reports from 2000 to 2022. This sentence should be clear and concise.
The author should consult a native speaker to check their grammar.
Overall, this is an interesting paper. After revision, I strongly recommend its publication in PLOS ONE.

**Response:** I have implemented the minor grammatical tweaks you recommended: First, the full forms of abbreviations in the title (e.g., source text (ST), target text (TT), Chinese-to-English (C-E)) have been included, as you suggested. Second, The sentence "This study aims to explore how translators function as mediators in this process by focusing specifically on the translation of modal verbs in Chinese government work reports from 2000 to 2022” has been revised for clarity and conciseness. It now reads: “This study explores how translators function as mediators through an examination of the Chinese-English translation of modal verbs in Chinese government work reports from 2000 to 2022.” To further ensure the grammar and phrasing meet the highest standards, I have consulted a native speaker for additional editing and proofreading. Thank you once again for your constructive suggestions, which have significantly improved the quality of the manuscript. Your attention to detail and insightful feedback is greatly appreciated.

**Response to Reviewer 5**

**Reviewer #5:** The authors investigate the role of translators as cultural mediators, using the translation of political texts as a case study and focusing on the rendition in English of modal verbs. While the study's intention is good, the methodology and findings are significantly flawed.
1) The quality of writing needs to be significantly improved to enhance the readers’ experiences.

**Response:** Thank you for your constructive feedback. In response to your comment, we have thoroughly revised the manuscript to improve the quality of writing and enhance readability. Significant efforts have been made to refine the language, improve clarity, and ensure that the arguments are presented in a more cohesive and scholarly manner. Additionally, we have expanded the manuscript by approximately 2,500 words to provide a more comprehensive and in-depth discussion of the methodology and findings. We believe these revisions will significantly enhance the readers’ experience and address the concerns raised, and we sincerely hope the updated version meets your expectations.

2) References may be needed when authors make profound claims like in the case of “Particularly, in political and official discourse, such as Chinese government work reports, the translator's role becomes even more critical. These documents are not merely informative texts but are vehicles for conveying policies, intentions, and national ethos.”

**Response:** Thank you for your valuable suggestion. In response to your comment, we have carefully added appropriate references to support statements such as “Particularly, in political and official discourse, such as Chinese government work reports, the translator's role becomes even more critical. These documents are not merely informative texts but are vehicles for conveying policies, intentions, and national ethos.” These references provide scholarly evidence to substantiate our claims and ensure that the arguments are well-grounded within relevant literature. We believe these additions strengthen the academic rigor of the manuscript and address the concern raised.

3) Psychological distance in translation: The explanation of this concept based on Nida, Venuti, and House does not expose the meaning intended. These authors’ writings do not explicitly focus on psychology, and to consider their discussions as such is unwarranted. If the authors interpret their writings as psychological distance, they are welcome to make their case.

**Response:** Thank you for your insightful comment regarding the conceptualization of psychological distance in our study. We appreciate the opportunity to clarify this important point. We would like to emphasize that our use of "psychological distance" and "psychological gap" is grounded in linguistic theory rather than psychological concepts. Following Halliday's (2010) framework, we examine how modal values reflect the strength of speaker tone and attitude. High-value modals express firm, affirmative attitudes, while low-value modals convey more tentative positions, with medium-value modals occupying an intermediate position on this continuum. Furthermore, as Hu & Tian (2018) demonstrate, modal verb usage reflects power relationships between speakers and listeners. High-value modals typically indicate social status inequality and authority, while medium and low-value modals suggest more equal relationships and mutual respect. In the context of our study, we analyze how translators modulate these modal values when rendering Chinese RWGs into English, effectively mediating the linguistic distance between source and target texts. This linguistic approach to psychological distance allows us to examine how translators systematically adjust modal values to bridge cultural and communicative gaps, making authoritative Chinese political discourse more accessible to international audiences while maintaining its essential message. Our analysis focuses on these linguistic mechanisms rather than psychological processes per se.

4) The discussion of the translator as a cultural mediator is seriously lacking citations from prominent scholars and works. Mention should be made of Bassnett and Lefevere, who coined the term “cultural turn” in translation.

**Response:** Thank you for your valuable suggestion. We appreciate your recommendation to include references to Bassnett and Lefevere’s seminal work on the "cultural turn" in translation studies. In response, we have incorporated relevant citations and discussions in the revised manuscript. Specifically, we now highlight the perspective that gained prominence with the "cultural turn," as argued by Bassnett and Lefevere (1990, 1998), emphasizing that translation operates as a form of cultural negotiation rather than mere linguistic transfer. Additionally, we reference Bassnett (2011), who underscores the translator’s role as a cultural mediator, bridging linguistic, cultural, and ideological gaps through conscious and informed choices. These additions provide a stronger theoretical foundation for our discussion of translators as cultural mediators and align our study with established scholarship in the field. We believe these revisions address your concern and enhance the academic rigor of the manuscript. Thank you again for your insightful feedback.

5) The literature review is scaringly scanty. It does not critically engage with the various concepts under discussion here, and no link is established between the elements discussed. For instance, what is the connection between psychological distance, culture, and government work? Why is it important to connect these variables in this research? Where is the gap in the literature, and how does this study address that gap? This claim, “This literature review reveals a gap in the research concerning the specific role of translators in mediating psychological distance through the translation of modal verbs in Chinese government work reports,” is not substantiated; it is imaginary.

**Response:** Thank you for your constructive feedback regarding the literature review. In response to your comments, we have thoroughly revised and expanded this section to provide a more systematic, comprehensive, and critical engagement with the key concepts discussed in the study. The revised literature review now explicitly addresses the connections between psychological distance, culture, and government work, highlighting their relevance to this research. Additionally, we have clarified the research gap by substantiating the claim that the role of translators in mediating psychological distance through modal verb translation in Chinese government work reports has been underexplored. The word count for this section has been increased from 562 to approximately 1,000 words to ensure depth and clarity. We hope these revisions address your concerns.

6) Structure of the paper: First, the research questions should come after the literature review. They should not be placed at the end of the introduction. Second, the theoretical foundation should precede the literature review. The theoretical framework should have its own choice rationale and, potentially, a gap that needs to be filled. The study needs to contribute to the theoretical framework to valorize its usage in this study.

**Response:** Thank you for your thoughtful and constructive feedback regarding the structure of the paper. In response to your suggestion, we have relocated the research questions from the introduction to the literature review section, as per your recommendation. This adjustment ensures a more logical flow and aligns the research questions with the theoretical and conceptual discussions. Regarding your suggestion to position the theoretical foundation before the literature review, we appreciate your perspective. However, in corpus-based empirical studies, it is common practice in our field to place the theoretical foundation within the research design section. This allows the theoretical framework to be directly connected to the corpus, methodology, and analytical procedures, ensuring coherence between theory and practice. In this revised structure, the theoretical foundation is introduced after the corpus description and before the research methodology, maintaining clarity and relevance. We hope these revisions address your concerns and improve the overall structure and rigor of the manuscript.

7) Methodology: There are several issues with the methodology and design of this study:

- There is a need to justify why the English corpus has more tokens than the Chinese corpus

**Response:** Thank you for your insightful comment regarding the discrepancy in token counts between the Chinese and English corpora. We appreciate the opportunity to clarify this point. The difference arises from the tokenization process applied to the Chinese texts. Unlike English, where words are naturally segmented, Chinese requires tokenization to separate characters into meaningful units (tokens) that approximate the concept of words in English. For example, the phrase "Report on the Work of the Government" consists of 7 English words but corresponds to 6 Chinese characters, which are tokenized into 3 tokens: "政府" (government), "工作" (work), and "报告" (report). This methodological approach ensures comparability between the two corpora. We have clarified this in the revised manuscript.

- Why was Halliday’s classification chosen? What other classifications, if they exist, are not suitable for this study?

**Response:** Thank you for your insightful question regarding the choice of Halliday’s classification for this study. I selected Halliday’s (2010) framework because it provides a robust and systematic approach to analyzing modal verbs based on their high, medium, and low modal values, which directly reflect the strength of a speaker's tone and attitude. This classification aligns well with the objectives of our research, as it allows us to examine how translators modulate modal values to mediate the psychological distance between the source text (ST) and target text (TT). Specifically, Halliday’s framework captures the continuum between negation and affirmation, with lower modal values closer to negation and higher values closer to affirmation, offering a nuanced lens for analyzing shifts in modality. Additionally, Hu and Tian (2018) extend Halliday’s framework by linking modal values to power dynamics between speakers and listeners. High-value modals often indicate authority and inequality, while medium and low-value modals suggest respect and equality. This perspective is particularly relevant for analyzing the Chinese RWGs, where modal verbs play a critical role in conveying authority and intent. We hope this explanation addresses your concern.

- Some concepts need to be explained or reiterated here, including “operational norms” and “psychological distance.”

**Response:** Thank you for your valuable suggestion regarding the clarification of key concepts. In response, we have revised the manuscript to provide clear definitions and reiterations of terms such as “psychological distance,” “cultural mediator,” and “operational norms” at appropriate points in the text. These additions ensure that readers can fully grasp the theoretical and practical implications of these concepts as they pertain to our study. We believe these revisions address your concern and improve the clarity and accessibility of the paper. Thank you again for your constructive feedback.

- What are “representative modals verbs,” and how were they determined?

Response: Thank you for your important question regarding the term “representative modal verbs.” We apologize for the lack of clarity in the original manuscript. In the revised version, we have specified that the “representative modal verb” refers to the Chinese modal verb “yao,” which appears consistently across all 23 texts in the corpus. This prevalence makes it highly representative for examining patterns of modal verb usage and translation strategies. We appreciate your feedback and believe this clarification strengthens the paper.

- There are also issues of reliability and validity in this methodology. Were the judgments made regarding psychological distance or the meaning of modal verbs in the corpora vetted by a third party? How? Was there a criteria or a set of guidelines to follow?

Response: The corpus-based methodology offers significant advantages in translation studies, particularly when analyzing large-scale texts. Firstly, it allows for the systematic analysis of large volumes of text, enabling researchers to uncover patterns and trends in translation strategies (Laviosa, 2002). Secondly, this method provides empirical data support, making it particularly suited for studying specific linguistic features, such as the translation of modal verbs (Liu et al., 2023). Additionally, corpus-based approaches facilitate comparative analyses across languages and genres, helping researchers understand how translators mediate between linguistic and cultural differences, thereby enhancing the effectiveness of cross-cultural communication (Hu, 2011). These features highlight the indispensable role of corpus-based methods in advancing research in translation studies. (I’ve added these sentences into the revised manuscript.)

This study, utilizing a parallel corpus of translations of modal verbs in the Chinese *Report on the Work of the Government* (2000–2022), ensures both breadth and representativeness with a total of 536,948 tokens. By extracting parallel concordances with modal verbs as node words, the research objectively analyzes trends and shifts in translation strategies and modal values, emphasizing the translator's mediation between linguistic and cultural differences. Grounded in Halliday's (2010) and Jiang & Yang's (2013) classification of modal verbs, the analysis adheres to scientifically established and consistent guidelines. The study follows a systematic process, including frequency statistics, modal value classification, and year-on-year analysis, with data that is verifiable and replicable. The strength of corpus-based methods lies in their ability to provide empirical, data-driven insights, avoiding the subjectivity of small-scale studies and enabling a comprehensive understanding of translation strategies and cross-cultural communication.

- What is the background of the translators who extracted the modals and their translations? How proficient are they in English and Chinese? How could their being Chinese or English native speakers influence their judgments, and how was this mitigated in the research?

**Response:** Thank you for your thoughtful comments regarding the background of the translators and the potential influence of linguistic proficiency and native speaker status on the judgments made in this research. I would like to provide clarification on these points: First, the Chinese Report on the Work of the Government (RWG) is translated by the Central Compilation and Translation Bureau, specifically the Central Translation Office, which operates under the authority of the Chinese government. The translators involved in this process are highly qualified professionals employed by the government, ensuring the accuracy and consistency of the translations. Second, the extraction, statistical analysis, and interpretation of the data in this study were conducted solely by me. I am an Associate Professor at Shanghai Lixin University of Accounting and Finance, with a PhD from Shanghai Jiao Tong University and a Master’s degree from Swansea University, UK. Furthermore, I have two years of research experience at the University of Texas at Dallas (UT Dallas) and have published articles in peer-reviewed English journals such as *Frontiers in Psychology, Heliyon*, and *Corpora*. As a native speaker of Chinese with extensive academic training and professional experience in English, I am confident that my proficiency in both languages is sufficient to ensure the reliability and validity of this research.

To mitigate potential biases, the analysis was grounded in objective, corpus-based methodologies, and all data extraction and classification followed established linguistic frameworks, such as Halliday's (2010) classification of English modal verbs and Jiang & Yang's (2013) classification of Chinese modal verbs. This systematic approach minimized subjective interference and ensured that the findings are robust and replicable.

I hope this explanation adequately addresses your concerns. Thank you again for your valuable feedback.

- The study is based on the assumption that translators consciously mediate between source and target text. What proof is there to show this was the case with these translations? Did government translators consciously mediate between ST and TT? If so, how do the authors know?

**Response:** Thank you for your comment regarding the assumption that translators consciously mediate between the source text (ST) and target text (TT). This is supported by evidence from multiple perspectives. First, the translation data reveals noticeable adjustments, such as translating high-value Chinese modal verbs based on context. These deliberate modulations indicate efforts to adapt the tone and modality to align with international norms and audience expectations. Second, statements from key figures in the Central Compilation and Translation Bureau highlight this intentional mediation. Lu Min described translating the Chinese RWGs as “a serious political task” requiring “fidelity and effectiveness” (Lu, 2002), while Huang Youyi emphasized principles like tailoring translations to international readers’ thinking habits and information needs (Huang, 2004). Finally, as a scholar with expertise in political and diplomatic discourse, I designed this study based on insights from prior research and supplemented it with systematic corpus-based analysis to verify these patterns. Together, these points demonstrate that government translators consciously mediate between ST and TT to ensure accurate, effective, and culturally adaptive translations.

- What criteria were used to classify models into the high and low levels? And what effort was made to ensure that all translators classified the modals the same way? Was the reliability checked?

**Response:** The study employs Halliday's (2010) and Jiang & Yang's (2013) frameworks for classifying modal verbs into different levels of modality (e.g., high, median, low), ensuring robust and established criteria are applied. These frameworks are well-acknowledged within linguistic research and provide clear distinctions based on semantic and functional properties of modal verbs. Institutional translation of the Chinese Report on the Work of the Government (RWGs), meanwhile, follows a rigorous multi-stage process. This process includes initial drafts, revisions by Chinese and foreign experts, collaborative discussions, and final reviews to ensure translation accuracy. Importantly, key linguistic elements such as modal verbs are standardized and discussed among experts to reduce individual translator subjectivity. Furthermore, the workflow ensures consistent and reliable translation of critical terms through collaborative efforts, particularly by addressing challenging areas with domain expertise. This institutional rigor guarantees that classifications of modality in the corpus are systematically verified and consistent, supporting the reliability of the data and analysis in the study.

8) Findings
Section 4.1
There is a fundamental flaw in the interpretation of “cultural mediator” here. It is unclear how this is achieved in the translation of modal verbs as outlined in this study. Authors state: “The translation of "Yao," the most frequent modal verb, provides a particularly insightful example of the translator's mediating function. As shown in Table 4, "Yao" is predominantly translated into four English modal verbs: will (36.11%), must (20.62%), need (19.16%), and should (17.94%). This distribution reveals the translator's nuanced interpretation of the source text's modal force, adapting it to suit different contexts and rhetorical purposes in the target language.” The problem is that this is an assumption since there is no evidence showing that the translator intended to culturally mediate these translations. Second, the authors need to prove that this was mediation by, for instance, showing how these modals are generally translated, or where they have been alternatively translated, for example, in non-government texts. Third, the assumption that all modals in the Chinese corpus were “mediated” seems to oversimplify cultural mediation in translation.
Explications and omissions are obvious translation techniques. How translators employ them here to “mediate culture” is unclear and has not been explained.

**Response:** Thank you very much for your comments. I acknowledge your astute observation regarding my interpretation of "cultural mediator" and agree that our terminology requires greater precision, and I have clarified it in the revised manuscript. Upon reflection, my analysis primarily demonstrates linguistic and pragmatic mediation rather than cultural mediation per se. The varying translations of "要(yao)" indeed reflect translators' linguistic choices within institutional and pragmatic constraints, rather than explicit cultural mediation. I concur that additional comparative analysis with non-governmental texts would strengthen our arguments about mediation patterns. My revised interpretation focuses on how translators navigate between source text modality and target language conventions within institutional translation frameworks, rather than assuming direct cultural mediation. I have modified our discussion to emphasize the pragmatic aspects of modal verb translation, acknowledging that translation choices are influenced by multiple factors beyond cultural considerations, including institutional guidelines, text type conventions, and communicative purposes in international discourse.

Section 4.2
The same argument above holds for this section. Nothing more than the author’s instinct justifies the claim that translators intentionally employed the English equivalent as “cultural mediators.”
Secondly, the lack of definitions in previous sections significantly hamper the understanding of this finding. Authors should have or should explain, high-value and low-value models. There is also a serious need to prove that the translation of modals into low-value modals in English is deliberate, not routine.

**Response：**Thank you for your valuable feedback. We appreciate the opportunity to clarify these points. First. As for Cultural Mediators: In the revised manuscript, we have further elaborated on the concept of translators as “cultural mediators.” Drawing on Hatim and Mason’s (1990) theory of cultural mediation and House’s (2015) concept of “cultural filtering,” we argue that the systematic modulation of modal values reflects translators’ role in bridging linguistic and cultural gaps. This is supported by corpus-based evidence of consistent patterns in modal shifts, as discussed in Sections 4.1 and 4.2. Second, High-Value and Low-Value Modals: The definitions of high-value and low-value modals are explicitly provided in the manuscript (e.g., Section 3.3 and 4.1). High-value modals (e.g., “要,” “必须”) express strong obligation or certainty, while low-value modals (e.g., “may,” “might”) convey weaker commitment. These definitions are grounded in Halliday’s systemic functional grammar and cultural-linguistic frameworks, emphasizing their relevance to the study. Third, Deliberate vs. Routine Choices: As stated in the methodology (Section 3.3), this study adopts a descriptive approach, focusing on the translation product rather than the translators’ intentions. Whether the observed shifts are deliberate or routine, our analysis highlights systematic patterns that reflect the translators’ mediating role in adapting Chinese political discourse for international audiences.

Factor Analysis
This section appears to repeat previous concepts without future explication. Plus, the authors introduce concepts like “positive and negative poles,”” Macro-level translation norms,” “international discourse system,” and “collectivist orientation of China,” which were not explained in the literature review and do not clearly align with anything else in the study. They are new and out of place.

**Response:** Thank you for your valuable feedback. We have carefully revised the section in question to address your concerns. Specifically, we have significantly rewritten and expanded the content to ensure clarity and coherence. Unrelated or unclear terms, such as “Macro-level translation norms” and “collectivist orientation of China,” have been removed to avoid confusion. The revised section now focuses on concepts directly relevant to the study’s objectives and aligns more closely with the theoretical framework and findings presented in the manuscript. We hope these revisions meet your expectations and improve the overall quality of the paper.
